# Typical pantothenate kinase-associated neurodegeneration caused by compound heterozygous mutations in *PANK2* gene in a Chinese patient: a case report and literature review

**DOI:** 10.3389/fneur.2023.1170557

**Published:** 2023-04-28

**Authors:** Yilun Tao, Chen Zhao, Dong Han, Yiju Wei, Lihong Wang, Wenxia Song, Xiaoze Li

**Affiliations:** ^1^Medical Genetic Center, Changzhi Maternal and Child Health Care Hospital, Changzhi, Shanxi, China; ^2^Department of Pediatrics, Changzhi Maternal and Child Health Care Hospital, Changzhi, Shanxi, China; ^3^School of Life Sciences, Shandong First Medical University and Shandong Academy of Medical Sciences, Jinan, Shandong, China; ^4^Medical Science and Technology Innovation Center, Shandong First Medical University and Shandong Academy of Medical Sciences, Jinan, Shandong, China; ^5^Obstetrics Department, Changzhi Maternal and Child Health Care Hospital, Changzhi, Shanxi, China

**Keywords:** *PANK2* gene, pantothenate kinase-associated neurodegeneration, whole exome sequencing, case report, review

## Abstract

Pantothenate kinase-associated neurodegeneration (PKAN) is a rare genetic neurodegenerative disorder with brain iron accumulation characterized as dysarthria, spasticity, cognitive impairment, parkinsonism, and retinopathy. PKAN is caused by biallelic mutations in the mitochondrial pantothenate kinase 2 (*PANK2*) gene. Herein, we report a 4-year-old patient with PKAN from a Han Chinese family, who presented with developmental regression, progressive inability to walk, and limb tremors. Neuroimaging demonstrated “eye-of-the-tiger” sign. Whole exome sequencing (WES) identified compound heterozygous mutations of c.1213T>G (p.Tyr405Asp) and c.1502T>A (p.Ile501Asn) in *PANK2* gene. In addition, a review of all known *PANK2* variants observed in reported PKAN patients was conducted, to improve understanding of the genotype-phenotype associations that occur in PKAN patients.

## Introduction

Pantothenate kinase-associated neurodegeneration (PKAN, MIM 234200) is a rare autosomal recessive disorder characterized by progressive iron accumulation in the basal ganglia and other regions of the brain, resulting in extrapyramidal movements such as parkinsonism and dystonia (1). On brain magnetic resonance imaging (MRI), abnormalities are restricted to the globus pallidus and substantia nigra in most cases of PKAN, with almost 100% having an “eye of the tiger” sign (2, 3). PKAN incidence is extremely low, reaching ~1–3/1,00,00,000 globally (4, 5), but the accurate prevalence of PKAN is unclear, particularly in the Chinese population.

The disease was first described in 1922 by two German physicians, Hallervorden and Spatz (6). In 2001, the cause of PNAK was determined to be a homozygous or compound heterozygous mutation in *PANK2* gene (MIM 606157), which is located on chromosome 20p13 and encodes a pantothenate kinase (7). The enzyme localizes to the mitochondria and phosphorylates pantothenate to synthesize coenzyme A (CoA) (8). Impaired activity of this pantothenate kinase may lead to increased levels of cysteine and its intermediate products in the basal ganglia. And meanwhile, iron accumulates with an unexplained mechanism. Cysteine can be chelated with iron and rapidly oxidizes itself. The resulting free cysteine disturbs energy metabolism and has a toxic effect on cell membrane synthesis, which can eventually result in central nervous system dysfunction (9, 10).

According to the age at onset, rate of progression, and severity of motor symptoms, PKAN can be classified into two subtypes: typical and atypical (2, 3). In typical PKAN, symptoms present within the first decade of life and usually progress rapidly, with loss of ambulation ~10–15 years later. In the atypical form, patients have an onset in the second decade, with slower progression and variable clinical features. Patients may still ambulate decades after disease onset.

In this study, we described the clinical phenotypes, biochemical features, and genetic findings of a Chinese patient with PKAN who had compound heterozygous mutations c.1213T>G (p.Tyr405Asp) and c.1502T>A (p.Ile501Asn) of *PANK2*. Furthermore, we review the clinical and genetic features of reported PKAN patients, to help understand the genotype-phenotype relationship of PKAN.

## Clinical report

**Figure 1:**
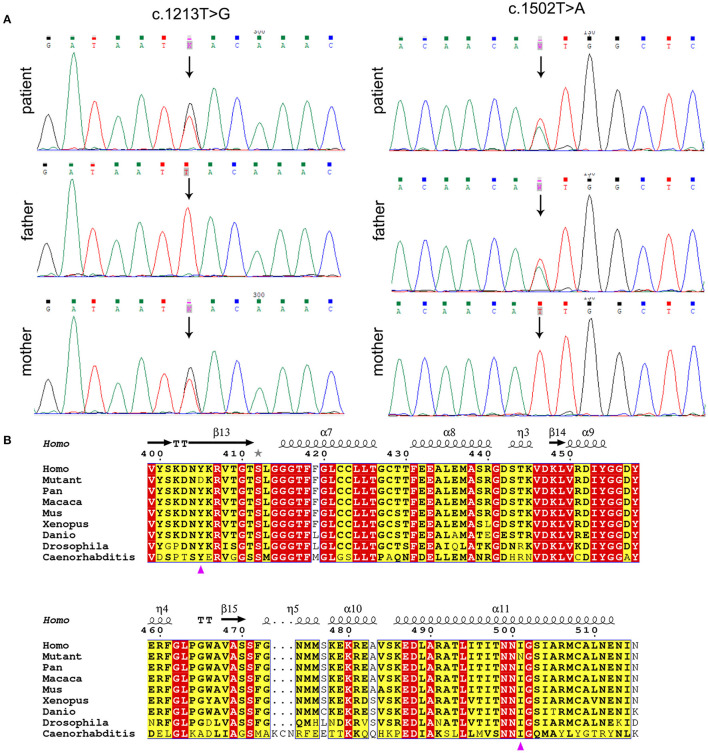
Clinical features and MRI examination of the proband. **(A)** Body scratches and bruise on the extremities by self-harm. **(B)** T2-weighted brain magnetic resonance image, showing the “eye-of-the-tiger” sign in the globus pallidus (arrow) at 4 years of age.

The proband, a 4-year-old female, was the first-born child of healthy and non-consanguineous Chinese parents. No history of genetic diseases exists in her family. The mother had no history of teratogenic pathogens or drug exposure during gestation. The birth weight was 3,770 g (75th percentile), and the birth length was 51 cm (50th percentile) at week 41 of gestation. At 18 months of age, the subject could walk with some aid but exhibited poor balance. At 3 years and 11 months of age, she exhibited developmental regression and could not walk, with progressive tremors in both the upper limbs and choreoathetosis. She had superimposed choreiform movements, mainly involving the distal upper limbs, and displayed involuntary self-injurious behavior (Figure 1A). Meanwhile, she exhibited progressive backward language formation and mental, intellectual disability retardation; she could not speak even simple words such as “baba” and “mama.” She seldom responded to language and was in a nearly semi-vegetative state. No feeding difficulties or dysphagia were found, and ophthalmological examination yielded normal results. Brain MRI at 4 years of age displayed a typical “eye-of-the-tiger” sign (Figure 1B). The child previously underwent rehabilitation therapy for several months; however, the effect was unsatisfactory. The couple underwent clinical genetic counseling and considered a prenatal diagnosis in a future pregnancy.

## Materials and methods

### Genetic analysis

A peripheral blood sample of the patient was collected. Genomic DNA was extracted using QIAamp DNA Mini Kit (Qiagen, China). DNA library preparation was performed following Illumina protocols, which included end repair, adapter ligation, and PCR enrichment. The amplified DNA was then captured using a Whole Exome Capture Kit (MyGenostics Inc., Beijing, China). Biotinylated capture probes were designed to tile all exons without repeating regions. The enriched libraries were sequenced for paired-end reads of 150 bp using Illumina HiSeq × Ten platforms.

After sequencing, the clean reads were aligned to the UCSC hg19 human reference genome using the Burrows-Wheeler Alignment tool. Duplicate reads were removed using Picard (http://broadinstitute.github.io/picard). Insertions, deletions, and SNP variants were detected and filtered using the Genome Analysis Toolkit. The identified variants were annotated using ANNOVAR and associated with the following databases: 1,000 Genomes, Exome Aggregation Consortium (ExAC), GnomAD Database, and Human Gene Mutation Database (HGMD). In addition, the identified variants were predicted using Amino acid substitutions were studied *in silico* to predict the pathogenic effect of the change using VarSome (https://varsome.com/), which utilizes multiple bioinformatic algorithms. Pathogenicity of the mutations was explored following the American College of Medical Genetics and Genomics guidelines (ACMG). Candidate variable sites were confirmed using Sanger sequencing of the patient and his parents. Target sequences were sequenced on an ABI 3730 genetic analyzer (Applied Biosystems, Foster City Carlsbad, CA, USA) and identified using Chromas 2.6.5 (Technelysium Pty Ltd, Australia).

### Literature review

A literature search of PubMed, MEDLINE, and EMBASE databases for articles published on PKAN on August 1, 2022, was conducted. The following keywords were used in the literature search: “pantothenate kinase-associated neurodegeneration” or “*PANK2*.” Pertinent articles found using the keywords and with definite molecular genetic features of PKAN were screened, and the clinical and genetic findings and prognosis conditions of patients were summarized.

## Results

**Figure 2:**
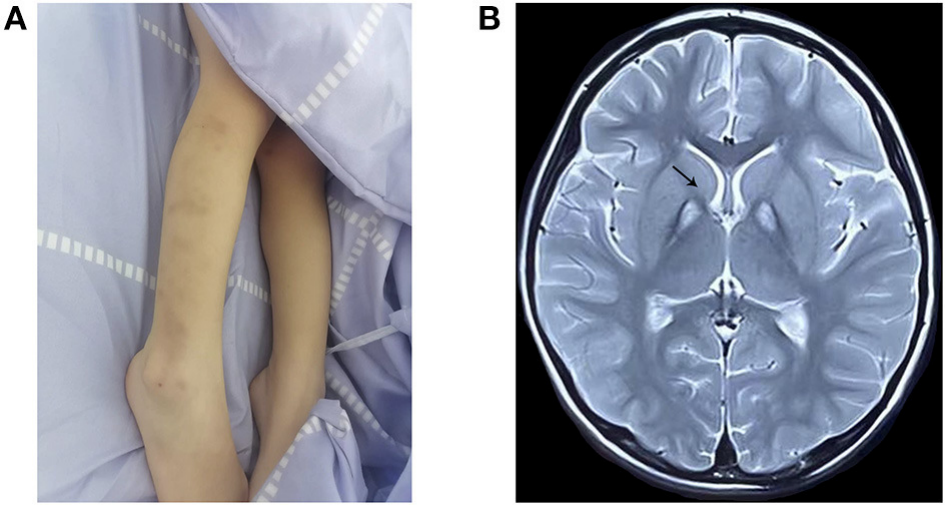
Molecular analysis of *PANK2* variants. **(A)** *PANK2* gene sequencing of the patient and his parents. The patient carried compound heterozygous PANK2 gene mutations of c.1213T>G and c.1502T>A; the father carried c.1502T>A, and the mother carried c.1213T>G. Arrow indicates the location of variants. **(B)** Multiple sequence alignment of PANK2 from different organisms. Residues are colored based on conservation. The white letters in red boxes represent the highest conservation-grade, black letters in yellow boxes represent the second-highest conservation grade, and no color indicates the least conserved grade. The variants p.Tyr405Asp and p.Ile501Asn are indicated by a purple triangle.

Whole exome sequencing (WES) data of the proband demonstrated two variants in *PANK2*, c.1213T>G (p.Tyr405Asp) and c.1502T>A (p.Ile501Asn) of transcript NM_153638.3, which were validated by Sanger sequencing (Figure 2A). The father was heterozygous for p.Ile501Asn and the mother was heterozygous for p.Tyr405Asp. Amino acid sequence alignment depicted that p.Tyr405Asp and p.Ile501Asn are highly conserved across different organisms, indicating that any non-synonymous change at positions 405 and 501 of PANK2 can be deleterious (Figure 2B). CADD values through VarSome were 26.7 for both two variants, also indicating the pathogenicity of both variants. According to ACMG classification, the two variants p.Tyr405Asp and p.Ile501Asn were predicted to be “likely pathogenic” (PP3_Strong+PM3+PM2_supporting) and “pathogenic” (PP3_Strong+PM3_strong+PM5+PM2_supporting). PAKN diagnosis in the proband was confirmed based on molecular genetics and available clinical findings.

## Discussion

In this study, we report a case of typical PKAN in a patient who presented with developmental regression, dystonia, progressive inability to walk, limb tremors, cognitive impairment, and dysarthria. Two compound heterozygous variants in exons 3 (p.Tyr405Asp) and 5 (p.Ile501Asn) were identified. This novel genotype was found for the first time in this study, as far as we know. Although p.Tyr405Asp was first reported in 2005 (3), no more cases have been reported, and this is the first report of this variant in China. Bioinformatic analysis revealed that the two variants were damaging, and the clinical presentation of the patient was compatible with PKAN. These findings indicate that p.Tyr405Asp and p.Ile501Asn variants are associated with PKAN pathogenesis. However, further functional studies are required to validate the pathogenicity of these variants.

The comprehensive clinical features and genetic analysis of 270 reported cases of PKAN (including the present case) are summarized in Supplementary Table 1. The majority of patients were Asian (96, 35.56%) or European (46, 17.04%). Among 266 patients with clear onset age, 167 cases (62.78%) showed the typical form, and 99 (37.21%), the atypical one. The mean age at presentation was 9.87 years (range 0.5–48). There is no sex-related difference in prevalence of PKAN, which were 52.34% (134/256) for males and 47.66% (122/256) for males, as well as in survival of PKAN (*p* = 0.8355). Most of the patients presented with dystonia (238/251, 94.82%), “eye-of-the-tiger” sign on MRI (246/258, 95.35%), gait disturbance (205/225, 91.11%), and dysarthria (174/198, 87.88%). Other common phenotypes included pyramidal signs (113/166, 68.07%), cognitive impairment (110/177, 62.14%), choreoathetosis (22/41, 53.66%), parkinsonism (68/127, 53.54%), ocular abnormalities (86/174, 49.43%), tremor (45/85, 52.94%), and developmental delay (25/72, 34.72%). In some patients (59/132, 44.70%), behavioral abnormalities developed, including psychosis, depression, hyperactivity, or obsessive-compulsive disorder. Only a few cases (*n* = 8) presented with seizures (11–16).

A total of 163 distinct *PANK2* variants were identified in these 270 patients (Supplementary Table 2), which were annotated based on transcript NM_153638.3. These 163 *PANK2* variants corresponded to 104 missenses, 31 frameshifts, 12 splice-sites, 11 non-senses and 5 large deletions. Variants were unevenly distributed throughout *PANK2* gene but were mainly concentrated in the exons (147/163, 90.18%), but particularly concentrated in exon 2 (27.61%), 3 (15.34%), and 4 (13.50%). One hundred and nine variants (66.87%) were observed only once or twice, indicating a high genetic heterogeneity among PKAN patients. The three most prevalent variants were p.Gly521Arg (5.91%), p.Asn404Ile (4.07%), and p.Thr528Met (3.88%), but have never been reported in the Chinese population. In addition, 114 out of 163 variants were absent in the Chinese population, and 40 variants were unique to the Chinese population. The six most prevalent variants in China, including p.Glu149Ter, p.Asp324Tyr, p.Asp378Gly, p.Asp452Gly, p.Ile501Thr and p.Phe519Leu, were only reported in Asia, including China, Korea, and Taiwan (4, 17–24). This finding indicates that the variants in *PANK2* are population-specific.

The genotypes of the 270 PKAN patients were found to be highly heterogeneous, with more than 45% of the patients presenting with a unique genotype, and 56.67% (153/270) with homozygous genotypes. A high amount of consanguineous marriages (45/115, 39.13%) may have contributed to the homogeneity. However, in China, only 29.41% (15/51) of the patients carried homozygous mutations, with a lower rate of consanguineous marriage (1/21, 4.76%).

In the current study, null *PANK2* alleles were defined as those containing non-sense, frameshift, and/or canonical splice site variants. Patients were divided into two groups with different genotypes: (i) M/M (missense/missense) (*n* = 161) and (ii) M/N (missense/null) or N/N (null/null) (*n* = 109). The age at onset was significantly earlier in “N/N” or “M/N” group than “M/M” group (median = 7.18 years vs. 11.24 years, *p* = 0.0003), and the ratio of typical early-onset patients in “N/N” or “M/N” group was larger than that in “M/M” group (80/107 vs. 87/159, 0.0009). Although there was no difference in survival of PKAN between patients with N/N or M/N and M/M genotypes (*p* = 0.4122), the death age was slight younger in those with null variants (median = 12.64 years vs. 14.80 years). Moreover, incidence of most phenotypes of the individuals with genotype “N/N” or “M/N” was larger compared with that of “M/M” group (Table 1). Taken together, these results indicate that patients with null variants might have a relatively poor survival. In this study, the proband carried compound heterozygous missense variants and was grouped in “M/M” genotype class. She appeared normal at birth, and there were no concerns about her development until over 3 years of age. The patient presented with the typical clinical manifestations of PKAN and a relatively poor prognosis. The patient also showed involuntary self-injurious behavior, which was not observed in any other PKAN patients, and poses challenges to the treatment and care of these patients.

**Table 1:** Clinical features of PKAN patients.

**Clinical feature**	**Frequency in reported affected individuals with pathogenic PANK2 variants**			
	**M/M** ^a^	**N/N or M/N** ^a^	**Total**	**This study**
Eye-of-the-tiger	149/156 (95.51%)	97/102 (95.10%)	246/258 (95.35%)	+
Tremor	31/60 (51.67%)	14/25 (56.00%)	45/85 (52.94%)	+
Parkinsonism	40/81 (49.38%)	28/46 (60.87%)	68/127 (53.54%)	/
Developmental delay	14/46 (30.43%)	11/26 (42.31%)	25/72 (34.72%)	+
Pyramidal signs	59/95 (62.11%)	54/71 (76.06%)	113/166 (68.07%)	+
Choreoathetosis	12/23 (52.17%)	10/18 (55.56%)	22/41 (53.66%)	+
Dystonia	142/153 (92.81%)	96/98 (97.96%)	238/251 (94.82%)	+
Cognitive impairment	60/104 (57.69%)	50/73 (68.49%)	110/177 (62.14%)	+
Dysarthria	104/122 (85.25%)	70/76 (92.11%)	174/198 (87.88%)	+
Dysphagia	29/64 (45.31%)	24/34 (70.59%)	53/98 (54.08%)	-
Gait disturbance	123/135 (91.11%)	83/91 (91.21%)	205/225 (91.11%)	+
Abnormality of the ocular region	45/106 (42.45%)	42/69 (60.87%)	86/174 (49.43%)	–
Behavioral abnormality	43/95 (45.26%)	16/37 (43.24%)	59/132 (44.70%)	–

^a^M, missense allele; N, null allele, null alleles were defined as those containing non-sense, frameshift, or canonical splice site variants.

In summary, a novel genotype of *PANK2*, c.1213T>G and c.1502T>A, was identified in this study. These findings provide further information regarding the genetic variants that cause PKAN. The review section describes all known *PANK2* variants, thus providing a basis for exploring genotype-phenotype correlations of PKAN.

## Data availability statement

The original contributions presented in the study are publicly available. This data can be found here: NCBI, accession number SRR23634966.

## Ethics statement

The studies involving human participants were reviewed and approved by Clinical Research Ethics Committee of Changzhi Maternal and Child Health Care Hospital. Written informed consent to participate in this study was provided by the participants' legal guardian/next of kin. Written informed consent was obtained from the individual(s), and minor(s)' legal guardian/next of kin, for the publication of any potentially identifiable images or data included in this article.

## Author contributions

CZ and DH performed the experiment and patient follow-up. YT conceived and designed the experiment, conducted genetic data acquisition and interpretation, reviewed the published cases, and wrote the manuscript. CZ, LW, and XL performed in patient management. WS provided the clinical treatment guidance. YW analyzed the data and revised the manuscript. All authors have read and approved the final manuscript.
